# Syk inhibitor attenuates lupus in FcγRIIb^−^^/−^ mice through the Inhibition of DNA extracellular traps from macrophages and neutrophils via p38MAPK-dependent pathway

**DOI:** 10.1038/s41420-025-02342-x

**Published:** 2025-02-17

**Authors:** Kritsanawan Sae-khow, Awirut Charoensappakit, Kanyarat Udompornpitak, Wilasinee Saisorn, Jiraphorn Issara-Amphorn, Tanapat Palaga, Asada Leelahavanichkul

**Affiliations:** 1 Center of Excellence in Translational Research in Inflammation and Immunology (CETRII), Faculty of Medicine, Chulalongkorn University, Bangkok, Thailand; 2 Department of Clinical Microscopy, Faculty of Allied Health Sciences, Chulalongkorn University, Bangkok, Thailand; 3 Functional Cellular Networks Section, Laboratory of Immune System Biology, National Institute of Allergy and Infectious Diseases NIH, Bethesda, USA; 4 Department of Microbiology, Faculty of Science, Chulalongkorn University, Bangkok, Thailand; 5 Department of Microbiology, Faculty of Medicine, Chulalongkorn University, Bangkok, Thailand; 6 Division of Nephrology, Department of Medicine, Faculty of Medicine, Chulalongkorn University, Bangkok, Thailand

**Keywords:** Translational research, Phagocytes, Autoimmunity

## Abstract

Spleen tyrosine kinase (Syk), an important hub of immune signaling, is activated by several signalings in active lupus which could be interfered by Syk inhibitor but is still not completely evaluated in innate immune cells associated with lupus activity. Hence, a Syk inhibitor (fostamatinib; R788) was tested in vivo using Fc gamma receptor-deficient (FcγRIIb^−/−^) lupus mice and in vitro (macrophages and neutrophils). After 4 weeks of oral Syk inhibitor, 40 week-old FcγRIIb^−/−^ mice (a full-blown lupus model) demonstrated less prominent lupus parameters (serum anti-dsDNA, proteinuria, and glomerulonephritis), systemic inflammation, as evaluated by serum TNFa, IL-6, and citrullinated histone H3 (CitH3), gut permeability defect, as indicated by serum FITC dextran assay, serum lipopolysaccharide (LPS), and serum (1 → 3)-β-D-glucan (BG), extracellular traps (ETs) and immune complex deposition in spleens and kidneys (immunofluorescent staining of CitH3 and immunoglobulin G) than FcγRIIb^−/−^ mice with placebo. Due to the spontaneous elevation of LPS and BG in serum, LPS plus BG (LPS + BG) was used to activate macrophages and neutrophils. After LPS + BG stimulation, FcγRIIb^−/−^ macrophages and neutrophils demonstrated predominant abundance of phosphorylated Syk (Western blotting), and the pro-inflammatory responses (CD86 flow cytometry analysis, supernatant cytokines, ETs immunofluorescent, and flow cytometry-based apoptosis). With RNA sequencing analysis and western blotting, the Syk-p38MAPK-dependent pathway was suggested as downregulating several inflammatory pathways in LPS + BG-activated FcγRIIb^−/−^ macrophages and neutrophils. Although both inhibitors against Syk and p38MAPK attenuated macrophage and neutrophil inflammatory responses against LPS + WGP, the apoptosis inhibition by p38MAPK inhibitor was not observed. These results suggested that Syk inhibitor (fostamatinib) improved the severity of lupus caused by FcγRIIb defect partly through Syk-p38MAPK anti-inflammation that inhibited both ET formation and cytokine production from innate immune cells.

## Introduction

Systemic lupus erythematosus (SLE) is a chronic inflammatory autoimmune disease with heterogeneity in clinical presentations [1]. There is an increase in the frequency of Fc gamma receptor IIb (FcγRIIb) dysfunction polymorphisms in patients with SLE, ranging from 0.7–1% in European [2] and over 10% in Asian populations [2, 3]. The FcγRIIb receptor is the only inhibitory receptor that controls many immune features, including phagocytosis, proinflammatory cytokine production, and antibody responses. Indeed, FcγRIIb deficient (FcγRIIb^−/−^) mice demonstrate hyperactive immune responses and are particularly prone to SLE [3, 4]. Although the hallmarks of SLE are adaptive immune abnormalities that elevate circulating autoantibodies that are bound to self-antigens generating immune complexes deposition and organ damages [5, 6], various innate immune cells have been implicated in SLE pathogenesis through several mechanisms, including antigen presentation [7], proinflammatory cytokines [5], DNA extracellular traps (ETs: an important source of nuclear antigens) [8], that might pave the way for novel treatment strategies [9]. Additionally, the innate immune also performs pro-inflammatory responses against microbial molecules that have been transferred from the gut contents (leaky gut) [10, 11], partly caused by the intestinal immune complex deposition in the large gastrointestinal (GI) surface area. Although the obvious GI manifestations in patients with lupus are rare [12], immune complex deposition in the gut [13] and spontaneous endotoxemia (an indirect indicator of leaky gut) in lupus [14] is demonstrated. Among lupus exacerbating factors from several causes, including photosensitivity [15], viral infections [16], environmental toxins [17], leaky gut [18], and obesity [19]. Spleen tyrosine kinase (Syk) is a nonreceptor tyrosine kinase with crucial roles in innate immune cells, including macrophages and neutrophils [20, 21], through downstream signaling transduction from surface receptors, such as Dectin-1 [22], Toll-like receptors (TLRs) [23], and Fc gamma receptor (FcγR) [20]. Indeed, Syk is an important signaling factor for phagocytosis, reactive oxygen species (ROS) generation, proinflammatory cytokine production, and neutrophil extracellular traps (NETs), which might exacerbate lupus activities [8, 20, 24]. Unsurprisingly, Fostamatinib, an US FDA-approved SYK inhibitor, has been currently developed as therapies for several autoimmune diseases, including immune thrombocytopenia (ITP), rheumatoid arthritis (RA) [25], and preclinical study in SLE [26] the impacts on lupus of this commercially available drug might be interesting.

In this study, roles of the Syk inhibitor in lupus disease activity and toward innate immune cells (macrophages and neutrophils), in full-blown FcγRIIb^−/−^ lupus mice, an active lupus model with leaky gut-induced endotoxemia and glucanemia [10], were explored. The capacity of Syk inhibitor to attenuate lupus disease progression in FcγRIIb^−/−^ lupus mice might lead to further application in patients with SLE in the future.

## Results

### Oral Syk inhibitor attenuated inflammation in FcγRIIb^−/−^ lupus mice

**Fig. 1:**
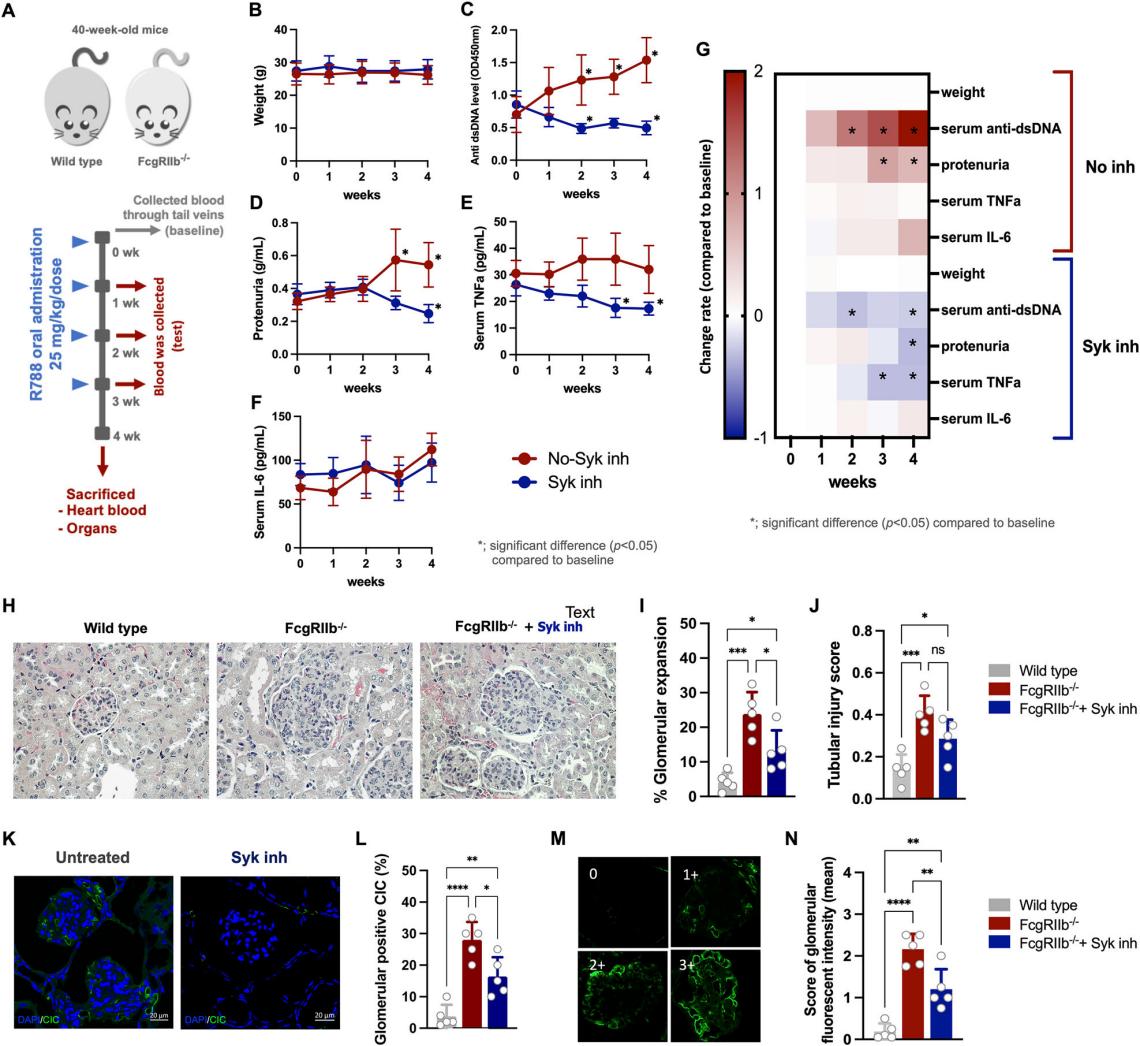
The effect of Syk inhibitor in FcγRIIb^−/−^ mice. Schema of the experiments (**A**) using wild type (FcγRIIb^+/+^) and knockout (FcγRIIb^−/−^) mice with Syk inhibitor (R788; Syk inh), once a week, via oral administration and tail vein blood collection with cardiac puncture under isoflurane anesthesia at sacrifice is demonstrated. The characteristics of FcγRIIb^−/−^ mice with and without Syk inh, as indicated by bodyweight, serum anti-dsDNA, proteinuria, serum TNFa, serum IL-6 (**B**-**F**) and representative these parameters in heatmap (**G**) are demonstrated. Additionally, the features of FcγRIIb^−/−^ mice (with Syk inh or vehicle) and wild type mice with vehicle, as indicated by renal histological score (percentage of glomerular expansion and tubular injury score) with representative pictures using hematoxylin and eosin (H&E) color (**H**-**J**), renal immunoglobulin deposition (CIC; circulating immune complex) in glomeruli (**K**, **L**) with the intensity score and representative fluorescent staining pictures (**M**, **N**) are demonstrated (*N* = 5/ group and time-point). *p < 0.05, **p < 0.01, ***p < 0.001, ****p < 0.0001; n.s., not significant.

To determine the efficacy of Syk inhibitor against SLE, R788 (fostamatinib) was orally administrated for four weeks to 40-wk-old female FcγRIIb^−/−^ mice (symptomatic lupus model) and age-gender matched wide type (WT) mice, to imitate the clinical situation (Fig. 1A). Indeed, 40-wk-old FcγRIIb^−/−^ mice showed lupus characteristics, including increasing levels of serum anti-dsDNA and proteinuria with proliferative glomerulonephritis in renal histology (Supplementary Fig. 1A-C), spontaneous elevation of serum cytokines, as evaluated by tumor necrosis factor alpha (TNFa) and interleukin-6 (IL-6), and an extracellular traps biomarker (serum citrullinated histone H3; CitH3) (Supplementary Fig. 1D-G). Additionally, lipopolysaccharide (LPS) and beta-glucan (BG), the major components of bacteria and fungi in gut microbiota, were observed in serum of FcγRIIb^−/−^ mice together with gut permeability defect, as tested by a fluorescein isothiocyanate-dextran (FITC-dextran) assay (Supplementary Fig. 1H-J). These data support active lupus with impaired gut permeability (leaky gut) and elevated microbial molecules in serum of 40-wk-old female FcγRIIb^−/−^ mice, supporting previous studies in patients and in mice [10, 11].

With Syk inhibitor, lupus characteristics and systemic inflammation in FcγRIIb^−/−^ mice were less prominent than the mice without inhibitor, as indicated by serum anti-dsDNA, proteinuria, and serum cytokines (TNFa but not IL-6) (Fig. 1C-F). The heatmap shows a summary of the alteration between FcγRIIb^−/−^ mice with versus without the inhibitor (Fig. 1G). Similarly, the mice with Syk inhibitor had less severe renal histology (Fig. 1H), as indicated by glomerular expansion (Fig. 1I) with a trend to reduced renal tubular injury (Fig. 1J) compared with vehicle-treated FcγRIIb^−/−^ mice. In accordance with these findings, renal immune-complex accumulation (Fig. 1K, L) and level of immune-complex deposition in glomeruli (Fig. 1M, N) were attenuated in Syk inhibitor-treated group. However, gut leakage parameters (serum FITC-dextran, serum endotoxin, and serum BG) did not show a significant difference between Syk inhibitor treatment and vehicle groups (Supplementary Fig. 1K).

### Syk inhibitor attenuated responses of FcγRIIb^−/−^ macrophages after activation by lipopolysaccharide (LPS) plus whole glucan particle (WGP)

**Fig. 2:**
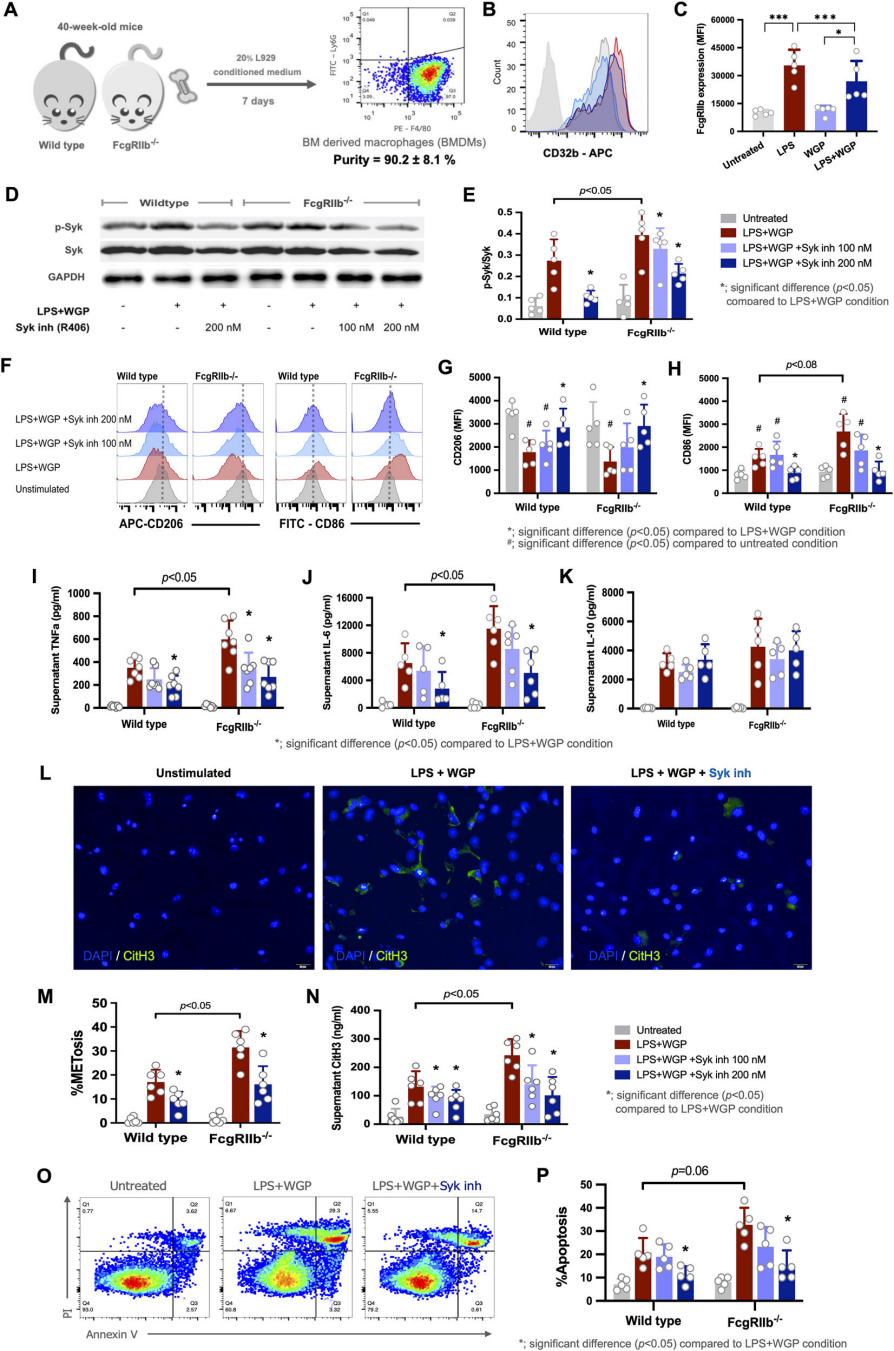
Effect of Syk inhibitor decreasing the inflammation in FcγRIIb^−/−^ BMDMs. Purity of the cells from bone-marrow-derived macrophages (BMDMs) as determined by F4/80 (**A**) and the expression of FcγR (CD32b) in wild type (WT) macrophages after stimulation by lipopolysaccharide (LPS) with and without whole glucan particle (WGP) (**B**, **C**) are demonstrated. Characteristics of BMDMs (WT and FcγRIIb^−/−^) after stimulation by LPS plus WGP (LPS + WGP) with and without a Syk inhibitor (Syk inh; R406) as indicated by Western blotting (**D**), the ratio of phosphorylated Syk/Syk (pSyk/Syk) from the Western blot analysis (**E**), markers of M1 pro-inflammatory macrophage polarization (CD86) and M2 anti-inflammatory macrophage polarization (CD206) with representative flow cytometry analysis pictures (**F**-**H**), supernatant cytokines (TNFa, IL-6, and IL-10) (**I**-**K**), macrophage extracellular traps (METs) as indicated by percentage of nuclear morphology alteration with representative co-staining pictures using DAPI (4′,6-diamidino-2-phenylindole; blue) and citrullinated histone 3 (citH3; green) staining, supernatant citH3 levels (**L**-**N**), and apoptosis as analyzed by flow cytometry using annexin V with propidium iodide (PI) (**O**, **P**) are demonstrated. The results were derived from 5 unrelated experiments. (*N* = 5/ group) *p < 0.05, ***p < 0.001; n.s., not significant.

Macrophages play a critical role in inflammation by producing various inflammatory mediators after recognizing several stimuli, including LPS and BG, by several receptors on the cell surface that activates several downstream signals, including several kinase enzymes [14, 27]. The extraction of bone marrow-derived macrophages (BMDMs) from femurs yielded more than 90% purity, as indicated by F4/80 macrophage indicator in flow-cytometry analysis (Fig. 2A). Because FcγRIIb (CD32b) was not detectable in FcγRIIb^−/−^ BMDMs, the effect of microbial molecules (LPS and WGP) on CD32b alteration was observed in WT BMDMs (Fig. 2B) using flow cytometry. In WT BMDMs, only LPS alone and LPS with whole glucan particle (WGP; the representative BG), elevated abundance of CD32b, whereas WGP alone did not elevate FcγRIIb. The reduced inhibitory FcγRIIb after LPS + WGP compared with LPS alone (bacterial molecule alone) might be responsible for the more prominent inflammation in LPS + WGP (combined the molecules from bacteria and fungi) (Fig. 2C). Because of the co-elevation of LPS and BG in the serum of mice with active lupus (Supplementary Fig. 1H, I), LPS + WGP with and without a Syk inhibitor (R406; the active form of Syk inh), but not LPS alone, were further tested.

In comparison with WT macrophages, LPS + WGP-activated FcγRIIb^−/−^ BMDMs demonstrated prominent protein expressions of both Syk and phosphorylated Syk (p-Syk), as evaluated by western blotting, with the more prominent M1 pro-inflammatory macrophage polarization (CD206^low^, CD86^high^) as analyzed by flow cytometry (Fig. 2D-H). In LPS + WGP-activated FcγRIIb^−/−^ BMDMs with Syk inh, the attenuated of p-syk with a shift toward M2 anti-inflammatory macrophage polarization (CD206^high^, CD86^low^) were demonstrated in a dose-dependent manner (Fig. 2D-H). In parallel, supernatant cytokines (TNFa and IL-6, but not IL-10) in FcγRIIb^−/−^ BMDMs were higher than WT BMDMs and Syk inh also attenuated these cytokines in a dose-dependent manner (Fig. 2I-K). The impact of LPS and WGP to induce PAD4 activation leading to extracellular traps (ETs) formation were well established [28]. Due to the well-known importance of DNA extracellular traps (ETs) in lupus progression [29], macrophage extracellular traps (METs) were measured by web-like structure representing cells with co-staining immunofluorescent between DAPI (4′,6-diamidino-2-phenylindole; blue color) nucleus staining and citrullinated histone 3 (citH3; green color) together with the levels supernatant citH3 measured by ELISA, as complementary method [30] (Fig. 2L-N). Indeed, macrophage extracellular traps (METs) of LPS + WGP-activated FcγRIIb^−/−^macrophages were prominent than LPS + WGP-stimulated WT cells. Notably, Syk inhibitor inhibited METosis in both WT and FcγRIIb^−/−^ BMDMs (Fig. 2L-N) with a reduction in apoptosis (annexin V and propidium iodide measured by flow cytometry) in a dose-dependent manner (Fig. 2O, P).

In accordance with these results, the hyper-activated Syk in LPS + WGP-stimulated FcγRIIb^−/−^ BMDMs with the stimulated WT cells might be associated with hyperinflammatory responses, as indicated by cytokine release, M1 polarization, cell apoptosis, especially METs. As expected, lists of the genes to explain METosis following the KEGG pathway of neutrophil extracellular traps was applied on a heat map (Supplementary Fig. 2). The results of LPS + WGP stimulated macrophages from WT and FcγRIIb^−/−^ mice demonstrated several genes that might be associated with NETosis, including NET stimulators (FcγR genes), NET downstream signals (PIK3K-ATK, phosphokinase C, and protein kinase C), chromatin de-condensation genes, and oxidative stress genes, as demonstrated which were reduced in Syk inh treatment condition consistency with the METosis result (Fig. 2L-N).

### Syk inhibitor attenuated inflammation through Syk-p38MAPK-dependent pathway

**Fig. 3:**
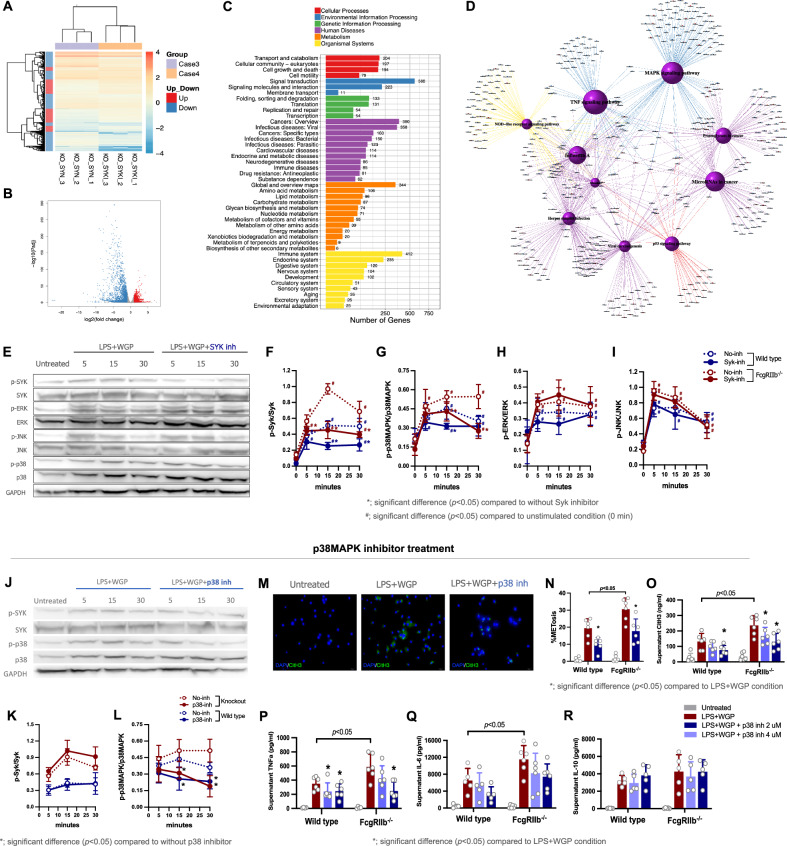
Transcriptomic and downstream signaling profiles of Syk-p38MAPK in FcγRIIb^−/−^ BMDMs stimulated with LPS+WGP. The characteristic of FcγRIIb^−/−^ macrophages after the activation by lipopolysaccharide plus whole glucan particle (LPS + WGP) without the inhibitor of Spleen tyrosine kinase (Syk-inh) (KO_SYN) and the LPS + WGP-activated FcγRIIb^−/−^ macrophages with Syk-inh (KO_SYKI), as indicated by transcriptome analysis through heat map (**A**), volcano plot (**B**), and KEGG pathway enrichment analysis with the bar chart (**C**) and as the network lines (**D**) are demonstrated. In the **D**, the diameters of the purple circles represent the number of annotated/associated genes, while the blue lines and the yellow to red lines represent the downregulated and up-regulated genes, respectively (the red color represents the highest intensity). The Western blotting (WB) analysis of LPS + WGP-activated FcγRIIb^−/−^ macrophages with and without Syk inh, as determined by phosphorylated (p-) and non-phosphorylated extracellular signal-regulated kinases (ERK), c-Jun N-terminal kinases (JNK), and p38 mitogen-activated protein kinase (p38MAPK), was demonstrated by the representative WB pattern from FcγRIIb^−/−^ macrophages, and the abundance WB score from both FcγRIIb^−/−^ and wild type (WT) macropahges (**E**-**I**) is also demonstrated. In parallel, the WB analysis of LPS + WGP-activated FcγRIIb^−/−^ macrophages with and without p38MAPK inhibitor (p38 inh) as indicated by p-Syk/Syk and p-p38MAPK /p38MAPK on the representative WB of FcγRIIb^−/−^ macrophages with the abundance WB score (**J**-**L**) is also shown. Then, the macrophage extracellular traps (METs) in LPS + WGP-activated macrophages with and without p38 inh as evaluated by nuclear morphology (DAPI; blue) and citH3 (FITC; green) as indicated by the representative immunofluorescent staining from FcγRIIb^−/−^ macrophages with the percentage of METosis (cell death from METs) together with supernatant citrullinated histone 3 (citH3) (**M**-**O**) are demonstrated. Additionally, the supernatant cytokines (TNFa, IL-6, and IL-10) are also demonstrated (**P**-**R**). The results were derived from 5 unrelated experiments. Notably, the representative pictures on WT cells (WB and fluorescent pictures) were not demonstrated due to the similar results between the WT and FcγRIIb^−/−^ macrophages. *p < 0.05.

To demonstrate the molecular mechanism of the Syk inhibitor in macrophages, transcriptomic analysis was performed. In comparison between LPS plus WGP-stimulated FcγRIIb^−/−^ BMDMs without Syk inhibitor (KO_SYN) and with Syk inhibitor (KO_SYKI), there were 1346 up- and 2199 down-regulated genes, as indicated by the heat map and the Volcano plot analysis (Fig. 3A, B) with the highest alteration in gene-related signal transduction (560 genes) (Fig. 3C). Meanwhile, the mitogen-activated protein kinase (MAPK) pathway and TNFa signaling were the top 2 pathway with the highest significant degrees of enrichment in the network analysis of pathway terms (Fig. 3D). According to the enrichment pathway in the group with Syk inhibitor, the major direction of the expressed genes was the downregulation of MAPK signaling pathway, as there were 91 up-regulated MAPK-related genes with only 23 down-regulated genes (Supplementary Fig. 3). Consistent with previous enrichment, the comparative between unstimulated and stimulated (LPS + WGP) conditions in FcγRIIb^−/−^ BMDMs showed the MAPK signaling pathway as the secondary highest degree of enrichment (Supplementary Fig. 4A). The subgroup analysis in stimulated condition between WT and FcγRIIb^−/−^ BMDMs (heat map) on the 2 top highest significant degrees of enrichment-related pathways (MAPK and TNF signaling) also showed the upregulation of MAPK and TNF signaling-related genes in FcγRIIb^−/−^ BMDMs (Supplementary Figs. 4B, 4C). These data implied the possible importance of MAPK in LPS + WGP activation in FcγRIIb^−/−^ BMDMs.

Based on the correlation of MAPK to other well-known molecules [31, 32], Syk might regulate MAPK signaling through extracellular signal-regulated kinases (ERK), c-Jun N-terminal kinases (JNK) and p38MAPK. Then, these molecules were explored in macrophages using western blot (WB) analysis (Fig. 3E). The expression of all molecules (Syk, ERK, JNK, and p38MAPK) were very low in unstimulated condition and prominently increase upon stimulation in both cell types (Fig. 3F-I). In FcγRIIb^−/−^ BMDMs without Syk inh, LPS + WGP more prominently activated phosphorylated Syk (p-Syk) compared to WT BMDMs (Fig. 3F). As such, in FcγRIIb^−/−^ macrophages with Syk inh, the decreased activation of Syk (p-Syk/Syk) and p38MAPK (p-p38MAPK/p38MAPK), but not ERK and JNK (p-ERK/ERK and p-JNK/JNK) were observed (Fig. 3F-I). The WT BMDMs and Syk inh also demonstrated significantly reduced the abundance Syk and p38MAPK activation (Fig. 3F-I). In parallel, p38MAPK inhibitor (Adezmapimod) was tested for the correlation between P38MAPK and LPS + WPG activation in macrophages. As such, the western blot analysis demonstrated that the p38MAPK inhibitor reduced p-p38MAPK abundance without an impact on p-Syk (Fig. 3J-L). Additionally, p38MAPK inhibitor also attenuated LPS + WGP-induced METs formation as determined by nuclear morphology (DAPI) colocalized with citH3 (FITC conjugated) (Fig. 3M-O) and the supernatant cytokines (Fig. 3P-R) in both FcγRIIb^−/−^ and WT BMDMs in a dose-dependent manner. Although the anti-inflammatory effect of p38MAPK inhibitor was similar to Syk inhibitor, the apoptosis inhibition by p38MAPK inhibitor was not observed (Supplementary Fig. 5). Despite a similar impact of LPS + WGP in both WT and FcγRIIb^−/−^ macrophages on inflammatory induction, Syk and inflammatory markers of FcγRIIb^−/−^ cells were more prominent and anti-inflammatory impact of Syk inh was more obvious in FcγRIIb^−/−^ macrophages.

### Syk inhibitor also reduced inflammatory responses of FcγRIIb^−/−^ neutrophils through Syk-p38MAPK dependent pathway

**Fig. 4:**
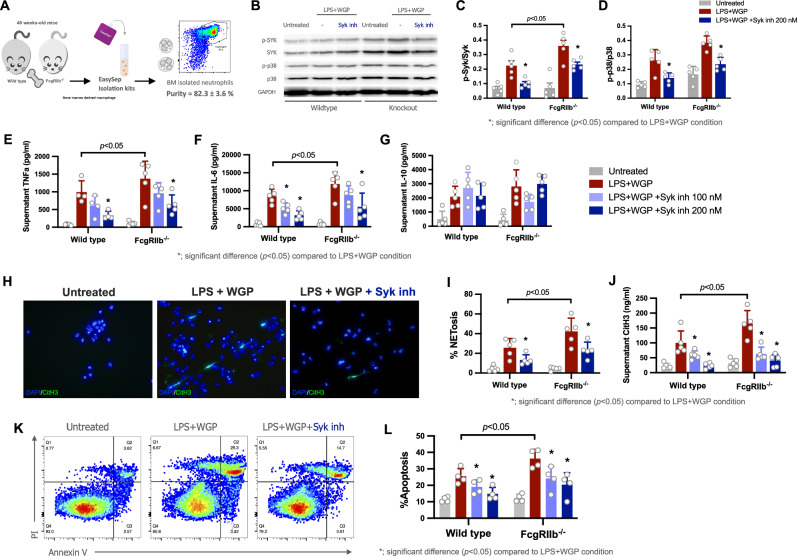
Attenuation of inflammation through Syk-p38MAPK in FcγRIIb^−/−^ neutrophils. Schema of the isolation and purity the bone-marrow-derived neutrophils as determined by Ly6g in flow cytometry analysis (**A**) and the characteristics of neutrophils from FcγRIIb^−/−^ and wild type (WT) after activation by lipopolysaccharide plus whole glucan particle (LPS + WGP) as indicated by phosphorylated and non-phosphorylated Spleen tyrosine kinase (p-Syk and Syk) and p38 mitogen-activated protein kinase (p-p38 and p38) as indicated by a representative Western blot (WB) of FcγRIIb^−/−^ neutrophils with the abundance WB score (**B**-**D**), supernatant cytokines (TNFa, IL-6, and IL-10) (**E**-**G**), neutrophil extracellular traps (NETs) as indicated by a representative immunofluorescent picture using DAPI nuclear morphology (blue) with citrullinated histone 3 (FITC-anti-citH3; green color) co-staining and the percentage score of the co-staining (**H**, **I**) together with supernatant citH3 levels (**J**), and apoptosis using flowcytometry analysis by annexin V with propidium iodide (PI) (**K**, **L**) are demonstrated. The results were derived from 5 unrelated experiments. Notably, the representative pictures on WT cells (WB and fluorescent pictures) were not demonstrated due to the similar results between the WT and FcγRIIb^−/−^ neutrophils. *p < 0.05.

A potential role of neutrophils in SLE pathogenesis and organ damage is well described, including neutrophil extracellular traps (NETs). Here, neutrophils were isolated from BM by a magnetic-based assay with an approximate purity of 82% (Fig. 4A). After LPS + WGP activation, FcγRIIb^−/−^ neutrophils demonstrated prominent increased p-Syk with similarly elevated p-p38MAPK when compared with LPS + WGP-activated WT neutrophils with Syk inh, as indicated by WB analysis (Fig. 4B-D). Likewise, LPS + WGP also elevated neutrophil supernatant cytokines, including TNFa, and IL-6, but not IL-10, NET formation (using colocalized citH3 with DAPI-web like structure morphology and supernatant citH3 levels), and apoptosis that were more prominent in FcγRIIb^−/−^ than WT neutrophils and were attenuated by Syk inh in both neutrophil types (Fig. 4E-L), similarly to the results of macrophages. With p38MAPK inhibitor in LPS + WGP activation, NETs and supernatant cytokines (TNFa and IL-6) were attenuated without an effect on supernatant IL-10 and apoptosis in both FcγRIIb^−/−^ and WT neutrophils (Supplementary Fig. 6). These findings implied a crucial role of Syk in stimulating FcγRIIb^−/−^ neutrophils through the Syk-p38MAPK axis, similar to macrophages.

### Syk inhibitor attenuates inflammation and extracellular traps (ETs) formation in FcγRIIb^−/−^ lupus mice

**Fig. 5:**
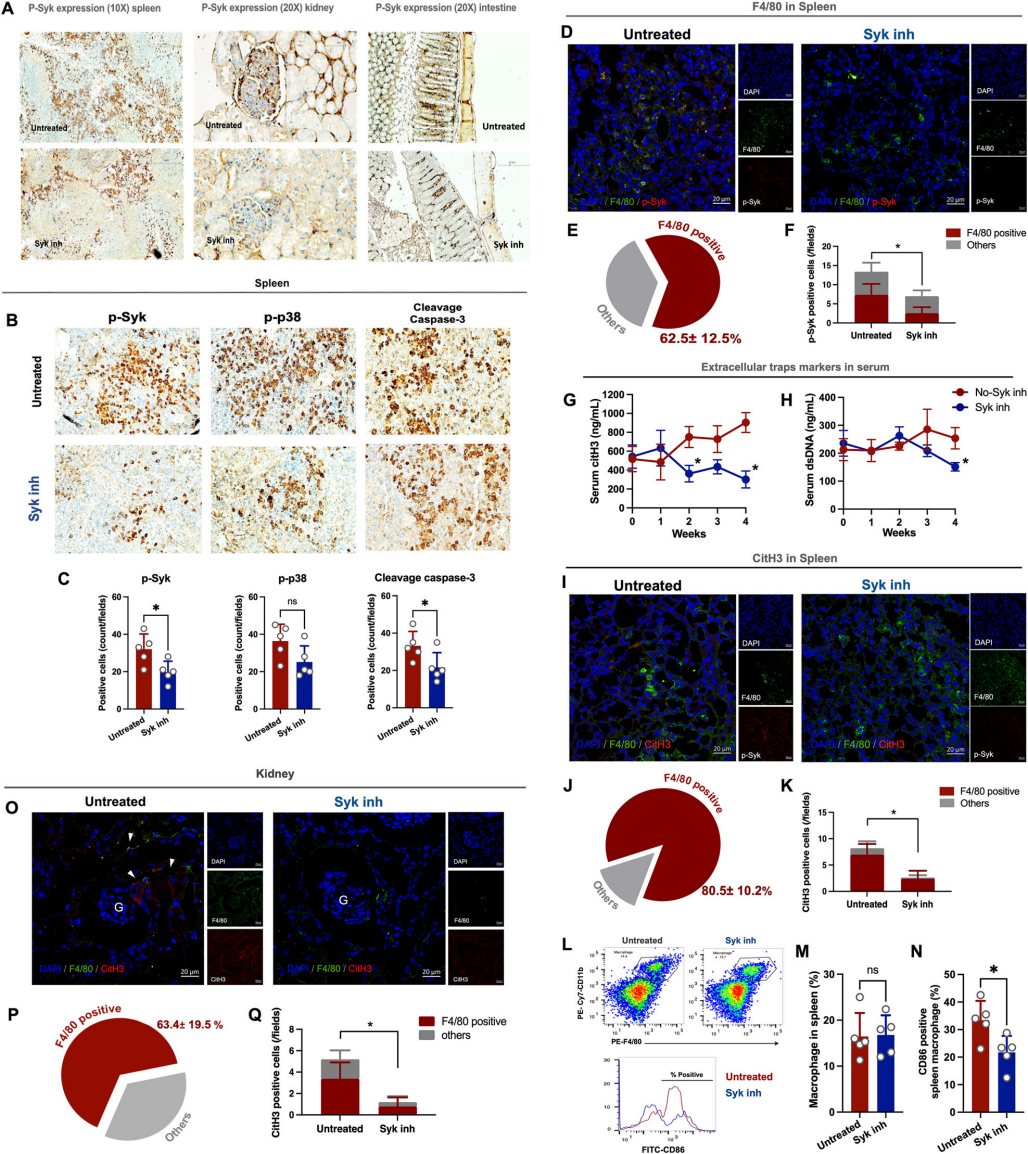
Reduction of Syk activation by Syk inhibitor to attenuate METs in spleen and kidney of FcγRIIb^−/−^ mice. Characteristics of 40-wk-old FcγRIIb^−/−^ mice with and without 4 wks of Syk inhibitor administration as indicated by the representative anti-phosphorylated Syk (p-Syk) immunohistochemistry picture in several organs (spleen, kidney, and colon) (**A**), the representative spleen immunohistochemistry for phosphorylated Syk (p-Syk), phosphorylated p38 MAPK (p-p38), and apoptosis (cleavage caspase 3) with the semiquantitative scores (**B**, **C**), the representative spleen immunofluorescence picture of p-Syk macrophages using the co-staining of F4/80 (green), p-Syk (red), and DAPI (blue) (**D**) with the scores in pie diagram (the proportion of F4/80 positive macrophage in red color is 62.5 ± 12.5%) (**E**) and the bar diagram of Syk positive cells (the red and gray colors represent p-Syk F4/80 positive macrophages and non-macrophages, respectively) (**F**), serum markers of extracellular traps (serum citH3 and dsDNA) (**G**, **H**), the representative pictures of spleen macrophage extracellular traps (METs) using the co-staining of F4/80 (green), citH3 (red) and DAPI (blue) (**I**) with the scores in pie diagram (the proportion of F4/80 positive macrophage in red color is 80.5 ± 10.2%) (**J**) and the bar diagram of CitH3-positive cells (the red and gray colors represent citH3 in macrophages and non-macrophages, respectively) (**K**), the representative dot plot-gated and histogram (flow cytometry) of the mature resident macrophages (F4/80^hi^CD11b^hi^) and CD86-positive macrophages in spleen (**L**) with the bar diagram of total macrophages and CD86-positive macrophages in spleen (**M**, **N**), and the representative pictures of renal macrophage extracellular traps (METs) using the co-staining of F4/80 (green), citH3 (red) and DAPI (blue) (**O**) with the scores in pie diagram (the proportion of F4/80 positive macrophage in red color is 63.4 ± 19.5%) (**P**) and the bar diagram of CitH3-positive cells (the red and gray colors represent citH3 in macrophages and non-macrophages, respectively) (**Q**) are demonstrated. The results were derived from 5 unrelated experiments. *p < 0.05; n.s., not significant.

Due to the Syk inh impact against pro-inflammatory responses and ETs formation of LPS + WGP-activated macrophages and neutrophils (Figs. 3, 4), Syk inh was further tested in mice using 4-wk-oral administration of Syk inh in 40-wk-old FcγRIIb^−/−^ mice, a symptomatic lupus model, as indicated by positive anti-dsDNA with proteinuria, leaky gut (FITC-dextran assay), endotoxemia, and glucanemia (Supplementary Fig. 1). As such, the reduced Syk activation in several organs (kidneys, spleens, and large intestines) in Syk inh-administered FcγRIIb^−/−^ mice with a prominent decrease in abundance in the spleen of Syk, p38MAPK, and apoptosis (cleavage activated caspase 3), as assessed by immunohistochemistry, was demonstrated (Fig. 5A-C). In contrast to WT mice, immunohistochemistry provided the slightly expression of Syk, p38MAPK activation and cleavage caspase 3 in spleen, while Syk inh also demonstrated tend to reduce these expressions (Supplementary Fig. 7).The co-staining of anti-F4/80 with anti-p-Syk immunofluorescent staining revealed that 62.5 ± 12.5% of the total Syk-positive cells at the white pulp of the spleen were macrophages, and Syk inh reduced Syk abundance in both macrophages (red-colored bar) and non-macrophages (gray-colored bar) (Fig. 5D-F).

Not only Syk abundance, Syk inh also decreased ETs formation, as indicated by reduced serum citH3 and serum ds-DNA after 2 and 4 wks of administration (Fig. 5G, H) and decreased METs formation in spleen, as determined by colocalized F4/80 with citH3 immunofluorescence (Fig. 5I-K). Interestingly, the total positive citH3 cells were mainly F4/80-positive cells (macrophages) at approximately 80.5 ± 10.2%, supporting the role of METosis in SLE pathogenesis (Fig. 5J). The F4/80^hi^CD11b^hi^ macrophages (mature resident macrophages) in the spleen (flow cytometry) were not decreased by Syk inh; however, CD86-positive macrophages (active pro-inflammatory M1 macrophage polarized cells) (the possible drivers of ET-related pathogenesis) [29], were decreased in FcγRIIb^−/−^ mice (Fig. 5L-N). In the kidney, METs (co-localization of F4/80 with citH3 immunofluorescence) mostly presented in the tubulointerstitial area but not in the glomeruli; however, renal METs were also reduced by Syk inh (Fig. 5O-Q). Parallelly, the prominent citH3 positive cells in kidneys were also macrophages (F4/80 positive) at approximately 63.4 ± 19.5% of all renal citH3-positive cells (Fig. 5P). Taken together, these results support the anti-inflammatory impact of Syk inh in FcγRIIb^−/−^ mice and might be useful for patients.

## Discussion

The inflammatory responses in active lupus are based on the deposition of circulating immune complexes (CIC) in several organs that induce chronic inflammation and tissue destruction [1, 9], with the emerging role of innate immunity [9, 33]. One of the situations during active lupus is the translocation of microbial molecules from the gut (LPS and BG) to the bloodstream, referred to as leaky gut or gut leakage, as demonstrated in symptomatic FcγRIIb^−/−^ mice and some patients [10], that provokes immune cells and enhances cell death (apoptosis), resulting in cell death-induced auto-antigen presentation, increased autoantibody production, elevated circulating immune complexes (CIC) deposition, and, finally, lupus disease exacerbation [10, 11]. Targeting innate immune cells in gut leakage environment, PRR signaling is the key mechanism that responds to pathogens by recognizing microbial molecules, especially LPS and BG, which mainly are toll-like receptor (TLR)-4 and Dectin-1, respectively. In addition, the crosstalk between activating FcγR (non-FcγRIIb) and the innate immune receptors (such as TLR-4 and Dectin-1) amplifies pro-inflammatory cytokine productions contribute to enhancing adaptive immunity associated with autoimmune disease by excessive inflammation [32], in FcγRIIb^−/−^ mice also promotes the pro-inflammatory responses in active lupus with endotoxemia and glucanemia from the leaky gut [33]. Naturally, the microbial molecules prime innate immune cells for the upcoming adaptive immunity partly through the up-regulation of several receptors, including the FcγR family, innate immunity control might efficiently attenuate adaptive immune responses in autoimmune disease [34]. Because Syk is a critical downstream signaling of FcγR (activated by the Fc portion of immunoglobulin) and is also a downstream signaling of TLR-4 and Dectin-1 [33], the presence of microbial molecules in the serum of FcγRIIb^−/−^ mice [20] might synergistically activate inflammation through Syk, as mentioned in several autoimmune diseases [34, 35]. Indeed, a Syk inhibitor (fostamatinib) is approved by the USFDA to be used for anti-inflammation in chronic immune thrombocytopenia [36] and possibly other autoimmune diseases (such as rheumatoid arthritis) along with post-COVID-19 pneumonia [37, 38]. Here, we demonstrate another possible mechanism of active SLE that might be suitable for the use of fostamatinib represented by FcγRIIb deficient-induced excessive inflammation, partly through p38MAPK in the innate immune cell (macrophages and neutrophils), due to the prominent Syk activation in FcγRIIb^−/−^ mice with endotoxemia and glucanemia. Although Syk inhibitors are beneficial in several autoimmune diseases [36] and some hyper-inflammatory situations [37, 38], Syk inhibitors might be even more effective in the lupus caused by FcγR polymorphism with leaky gut-induced endotoxemia and/or glucanemia.

Accordingly, the spleen tyrosine kinase (SYK) plays a crucial role in various signaling pathways of inflammation, especially in lupus dysregulated inflammation [33], due to CIC and microbial molecules from the gut [10]. Here, FcγRIIb^−/−^ mice with active lupus and leaky gut demonstrated profound inflammation with prominent Syk activation (immunohistochemistry analysis), especially in the kidneys, spleens, and intestines, implying the possible recognition of CIC, LPS, and BG through Syk signaling. Meanwhile, the absence of gut leakage in WT mice with the intact inhibitory FcγRIIb receptor resulted in lower inflammatory responses. Indeed, the inhibition of Syk activity is known to harness multiple downstream signaling pathways, which supported possible effectiveness of Syk inh against lupus, as indicated by i) the reduced Syk activation in several organs and lupus activity attenuation in FcγRIIb^−/−^ mice with fostamatinib (R788), and ii) a decrease in ETs and pro-inflammatory responses in FcγRIIb^−/−^ macrophages and neutrophils. Innate immune cells serve as sentinel cells, patrolling for defense against pathogens. Overwhelming of the inflammatory environment, whether sterile or non-sterile, develops chronic inflammation, as partly indicated by the impacts of ETs in macrophages and neutrophils during active lupus [39]. Although NETs have been extensively linked to poor outcomes in SLE [24, 40, 41], the roles of macrophage extracellular traps (METs) in lupus still need further investigation. In the spleens of FcγRIIb^−/−^ mice, more than 60% of macrophages demonstrated Syk activation, which mostly induced MET formation (co-staining of F4/80 and citH3) with prominent apoptosis. Meanwhile, Syk-activated macrophages were also prominent in the interstitial area of the kidneys of FcγRIIb^−/−^ mice. With the prominent Syk activation, it was non-surprising that Syk inhibitors could attenuate METs, macrophage apoptosis, and reduce inflammatory M1 macrophage status, along with lupus disease activities (proteinuria, dsDNA, and renal injury). While the Syk inhibitor attenuated the inflammatory responses from leaky gut–derived endotoxemia and glucanemia, the severity of leaky gut in FcγRIIb^−/−^ mice was not altered by the inhibitor [42, 43]. Perhaps, the longer administration of the Syk inhibitor more than 4 wks is necessary for improving gut permeability defects.

Roles of FcγRIIb were determined in WT macrophages (no FcγRIIb expression in FcγRIIb^−/−^ macrophages); FcγRIIb was dominantly expressed in WT macrophages after stimulation by LPS or LPS plus WGP (LPS + WGP), but not WGP alone, suggesting that FcγRIIb may be a compensatory mechanism to prevent the overwhelming responses [44, 45]. Perhaps, the crosstalk between inhibitory FcγRIIb and LPS might be one of the regulatory mechanisms to reduce hyper-inflammatory responses [14]. More mechanistic studies on this topic are interesting. Because of the co-presentation of LPS and beta-glucan in the serum of the FcγRIIb^−/−^ mice with active lupus, only LPS + WGP, but not LPS alone, was further tested in vitro. Accordingly, inhibitory activation through the activation of inhibitory FcγRIIb with either Syk or other activating receptors is dephosphorylated by SH2-containing inositol phosphatase (SHIPs) on downstream targets following the inhibiting signaling cascade [46]. As expected, Syk activation was predominant in LPS + WGP-activated FcγRIIb^−/−^ macrophages than the WT cells, which might be associated with the higher expression of MAPK and TNF signaling pathways, as demonstrated in our transcriptome analysis. In addition, LPS + WGP induced METs, NETs, inflammatory cytokines (TNFa and IL-6), and apoptosis more dominantly in FcγRIIb^−/−^ macrophages and neutrophils than the WT cells, which were attenuated by Syk inhibitors. As such, MAPK signaling is a key mediator of inflammatory mechanisms that have been classified into 3 subgroups, including ERK (classical), p38, and JNK (alternative). Elucidated signaling cascade of Syk-MAPK in activation of FcγRIIb^−/−^ macrophages and neutrophils by Syk inhibitors, p38MAPK was downstream signaling, not ERK and JNK (Western blot analysis), implying the importance of SYK-p38MAPK signaling in LPS + WGP-activated FcγRIIb^−/−^ macrophages.

Additionally, the SYK-p38MAPK signaling cascade is also demonstrated in the hepatocytes of the liver injury model [46]. Indeed, the role of p38MAPK signaling in autoimmune diseases has also previously been tested to ameliorate disease severity through various mechanisms [47–49]. Here, p38MAPK blockage also reduced inflammation in LPS + WGP-activated macrophages. While the impact of NETs-SYK and NETs-p38MAPK in SLE is well-known [50–53], data on the signaling of METs in lupus is still limited. Here, we highlight the possible implications of targeting ETs, especially METS, through the Syk-p38MAPK-dependent pathway in FcγRIIb^−/−^ lupus mice that might develop novel therapeutic strategies against active lupus. Because there are several underlying molecular mechanisms of lupus, tailoring treatment for patients concerning the possible molecular differences using the targeted drug might be one of the interesting futures of “personalized medicine” in lupus patients with FcγRIIb dysfunction polymorphism and/or leaky gut.

In summary, Syk activation was more prominent in FcγRIIb^−/−^ than WT mice, and Syk inhibitors (fostamatinib) effectively attenuated the severity of lupus characteristics (anti-dsDNA levels, proteinuria, and renal histology) in FcγRIIb^−/−^ mice. Additionally, Syk inhibitors downregulated inflammation (cytokine production and extracellular traps) in macrophages and neutrophils (more prominently in FcγRIIb^−/−^ cells than WT cells) in the Syk-p38MAPK-dependent pathway and were proposed as an interesting candidate for the treatment of active lupus, especially in patients with FcγRIIb dysfunction polymorphism. Exploring genetic background and/or gut leakage in lupus might be a new strategy for directly targeted treatment. More studies would be interesting.

## Supplementary information

Western Blot full lenght

Supplementat Figure 1-7

Supplementation Materials and Methods

## Data Availability

The datasets generated or analyzed during this study are included in this published article or available from the corresponding author on reasonable request. RNA-seq data have been deposited at SRA: PRJNA1133910 are publicly available as of the date of publication.
