# Supplementary material for: Syk inhibitor attenuates lupus in FcγRIIb−/− mice through the Inhibition of DNA extracellular traps from macrophages and neutrophils via p38MAPK-dependent pathway
Source: Cell Death Discov. 2025 Feb 17;11:63. doi: 10.1038/s41420-025-02342-x (PMC11832894; doi:10.1038/s41420-025-02342-x)
Supplement: Supplementary file 1 — Western Blot full lenght [file 41420_2025_2342_MOESM1_ESM.pdf]

Figure 2D
BMDM

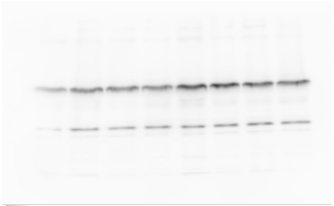

72 kDa pSyk

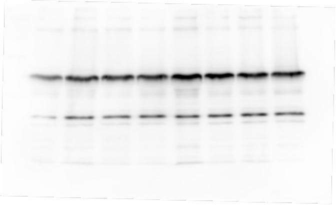

72 kDa Syk

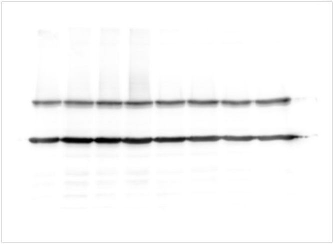

37 kDa GAPDH

Figure 3E
BMDM

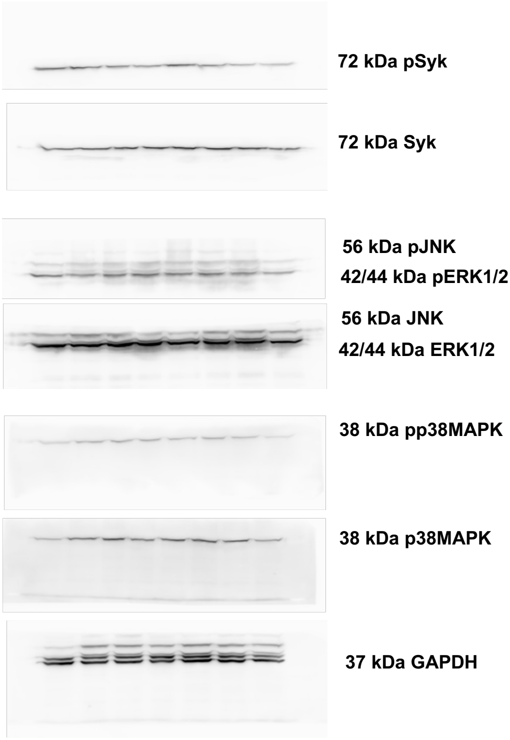

Figure 3J
BMDM

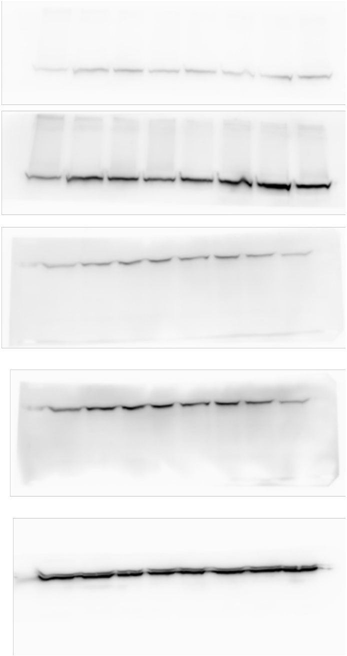

72 kDa pSyk

72 kDa Syk

38 kDa pp38MAPK

38 kDa p38MAPK

37 kDa GAPDH

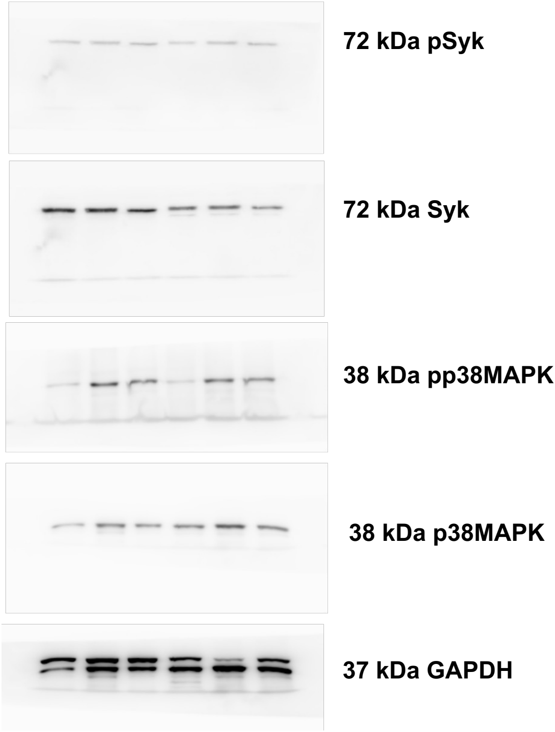

Figure 4B
Neutrophil
