# Supplementary material for: Syk inhibitor attenuates lupus in FcγRIIb−/− mice through the Inhibition of DNA extracellular traps from macrophages and neutrophils via p38MAPK-dependent pathway
Source: Cell Death Discov. 2025 Feb 17;11:63. doi: 10.1038/s41420-025-02342-x (PMC11832894; doi:10.1038/s41420-025-02342-x)
Supplement: Supplementary file 2 — Supplementat Figure 1-7 [file 41420_2025_2342_MOESM2_ESM.pdf]

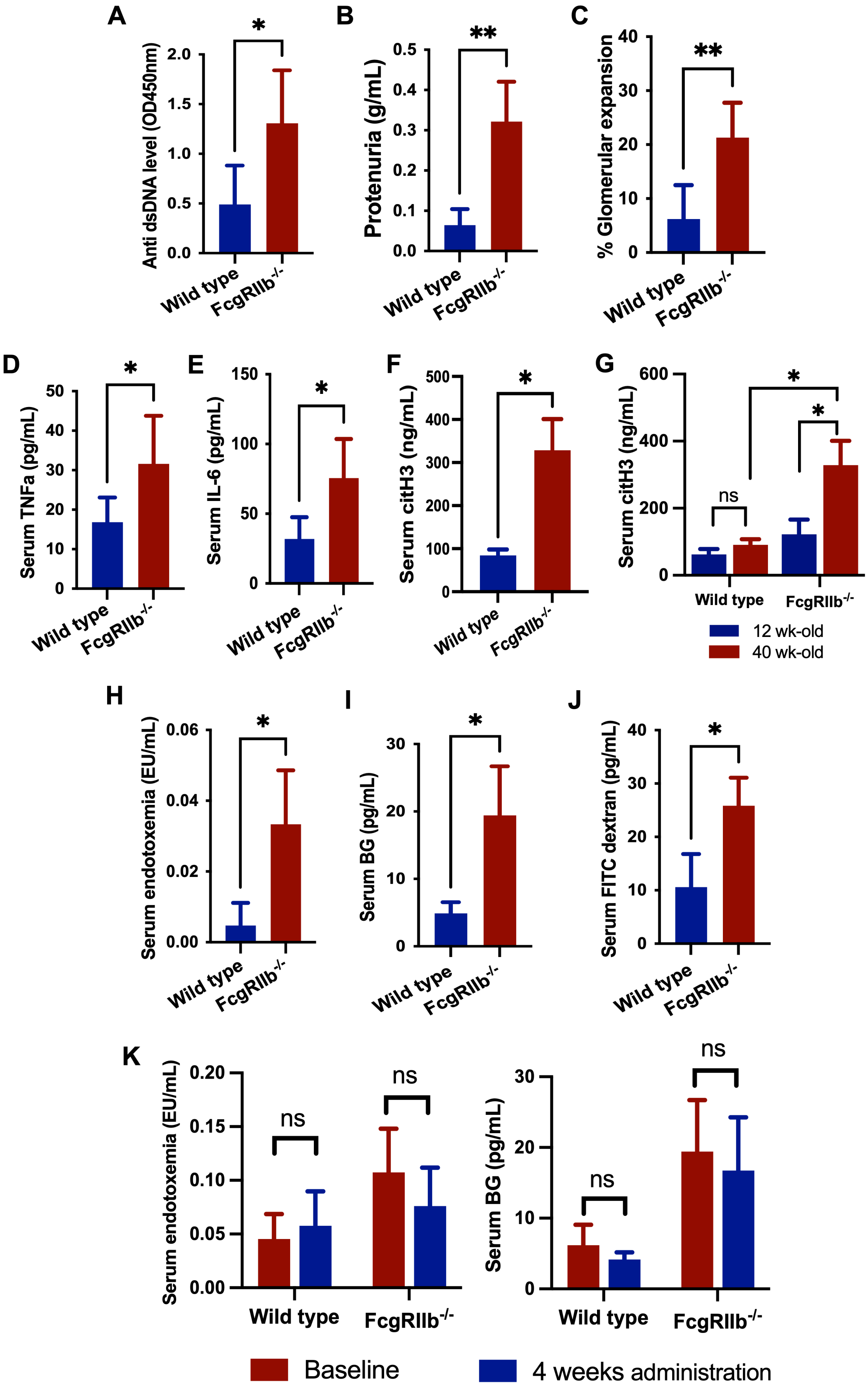

Supplement Figure 1: The characteristics of lupus were compared in 40-wk-old wild type (FcγRIIb$^{+/+}$; n=5)) and knockout (FcγRIIb$^{-/-}$; n=5) mice. Serum anti-dsDNA level (A), proteinuria (B), %glomerular expansion (C), serum TNFa (D), serum IL-6 (E), serum citrullinated histone H3 (CitH3) (F), comparison of serum citH3 between 12 and 40-wk-old mice (G), and markers of gut leakage; serum endotoxemia (H), serum BG (I), serum FITC dextran (J), and serum endotoxemia and BG after 4 weeks administration of Syk inhibitor compare with baseline.

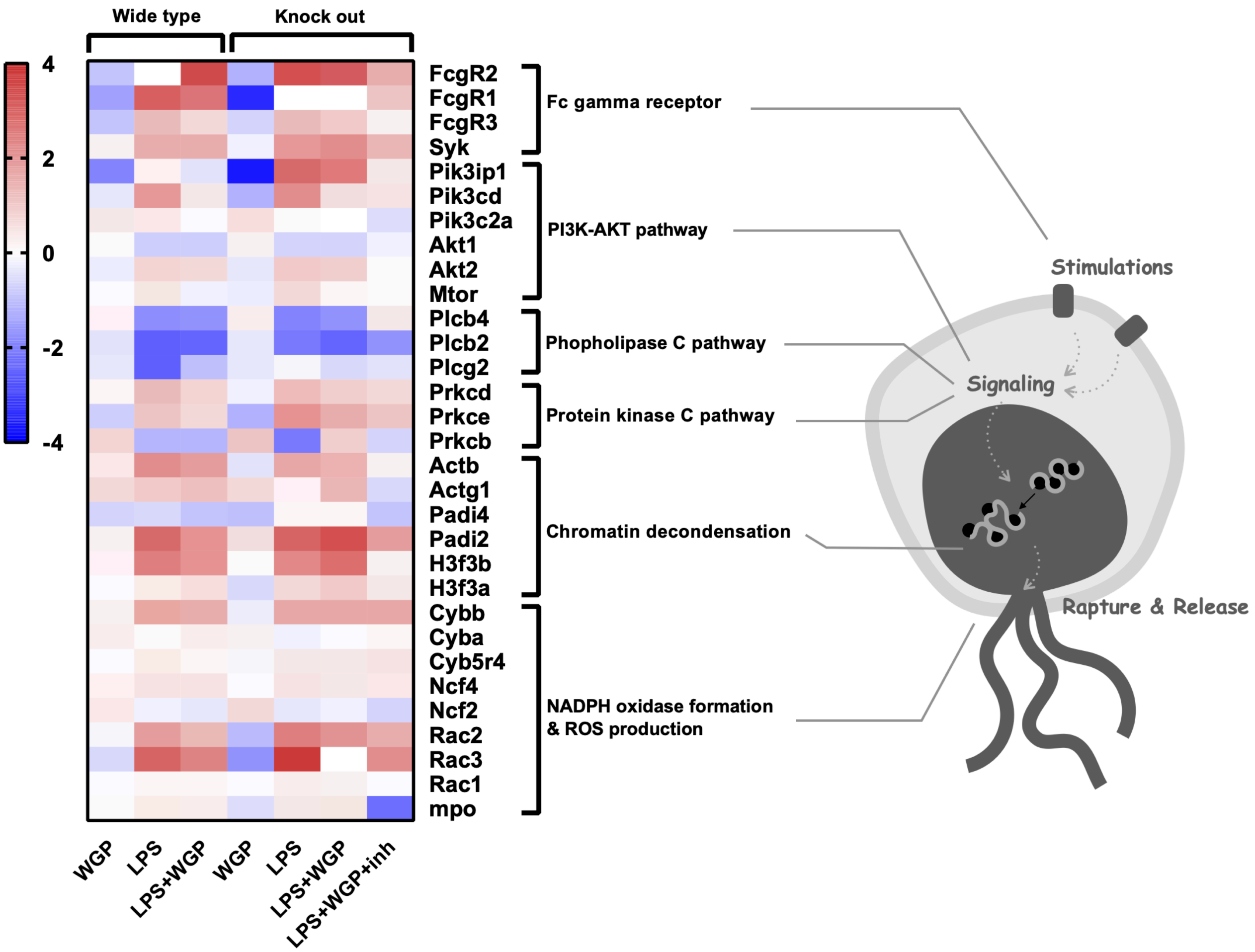

Supplement Figure 2: Heat map results of RNA sequencing in KEGG pathway (hsa04613) using extracellular traps related gene panel that compare between LPS+WGP stimulation of wild type (FcγRIIb$^{+/+}$) and knockout (FcγRIIb$^{-/-}$) BMDMs, and priming Syk inhibitor (R406) in FcγRIIb$^{-/-}$.

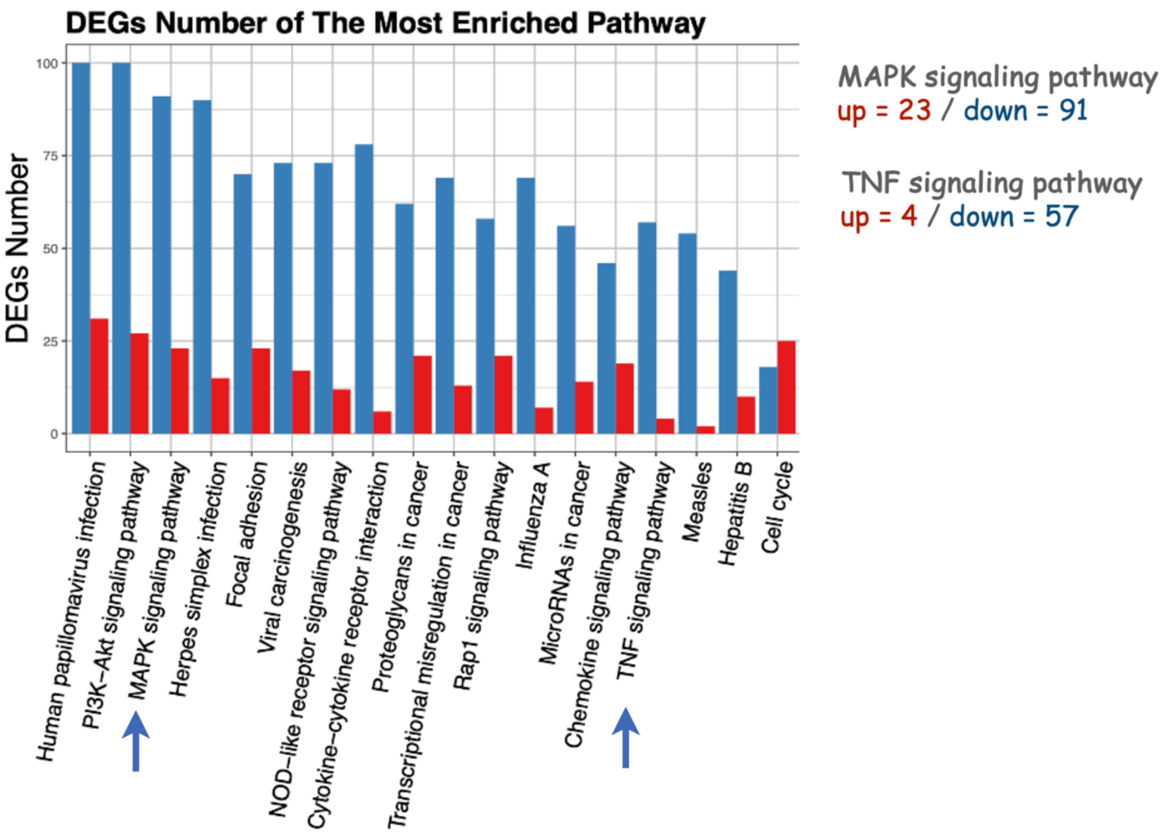

Supplement Figure 3: The MAPK and TNFa signaling were the top 2 highest significant degrees of enrichment pathway from KEGG database in Fc**γ**RIIb$^{-/-}$ BMDM with Syk inb. priming then stimulation with LPS+WGP compared to non-Syk priming with LPS+WGP stimulation condition which majority of genes were down-regulated expression (MAPK signaling pathway; 91 down-regulated and 23 up-regulated, TNFa signaling pathway; 57 down-regulated and 4 up-regulated).

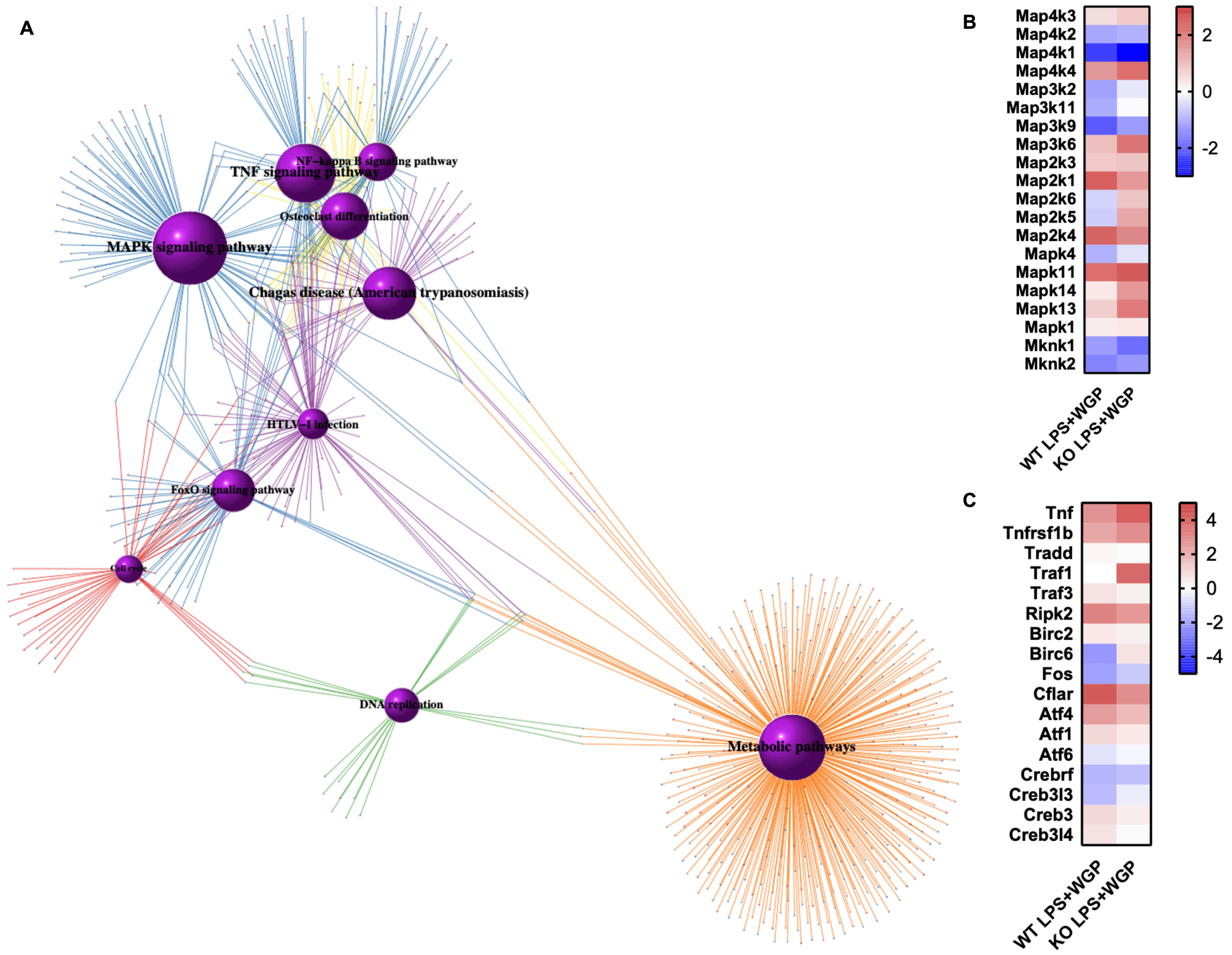

Supplement Figure 4: KEGG enrichment analysis of FcγRIIb$^{-/-}$ BMDMs (LPS+WGP combination compared to untreated group) (A). The up-regulated and down-regulated significant gene expression are defined by red and blue dots. The pathway's circle size represents the higher degree of top ten enriched pathways. The 6 color lines indicate functionals of enrichment, red for cellular processes, blue for environmental information processing, purple for human diseases, and yellow for organic system. Heatmaps were visualized in comparison between FcγRIIb$^{-/-}$ and WT BMDMs in LPS+WGP condition in MAPK pathway (B) and TNFa pathway (C).

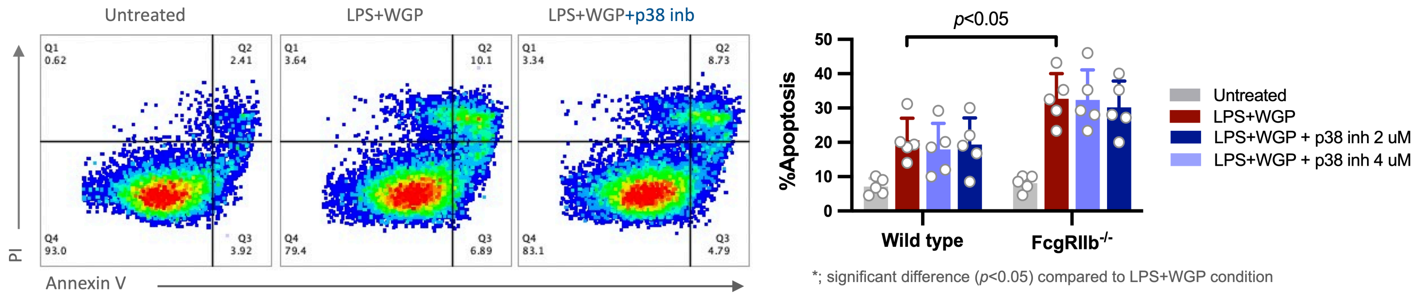

Supplement Figure 5: The effect of p38MAPK inhibitor in LPS+WGP stimulated WT and Fc**γ**RIIb$^{-/-}$ BMDM apoptosis characterized by flow cytometry analysis with anexin V and PI. *; significant difference (p<0.05) compared to LPS+WGP in each group.

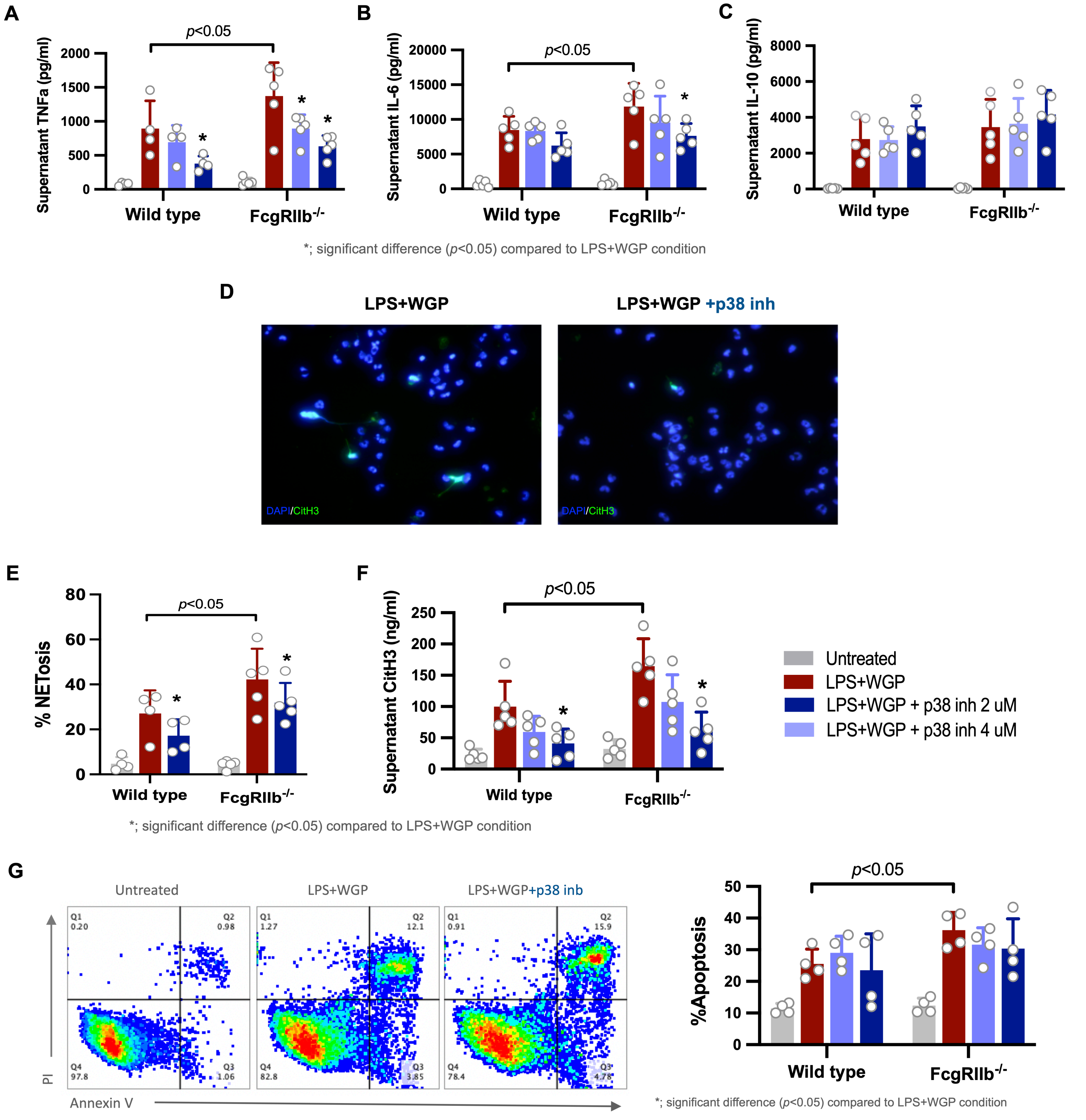

Supplement Figure 6: p38MAPK inhibitor (Adezmapimod) priming WT and FcγRIIb$^{-/-}$ neutrophils before LPS+WGP stimulation. The comparison of supernatant cytokines: TNFa (A), IL-6 (B), and IL-10 production (C). Neutrophil extracellular traps as defined by immunofluorescence (D), co-staining of DAPI (blue) and FITC-anti-citH3 (green), calculated in percentage of NETosis (E) together with level of supernatant citH3 (F). Apoptosis assay evaluated by flow cytometry with anexin V and PI. *; significant difference ($p<0.05$) compared to LPS+WGP in each group.

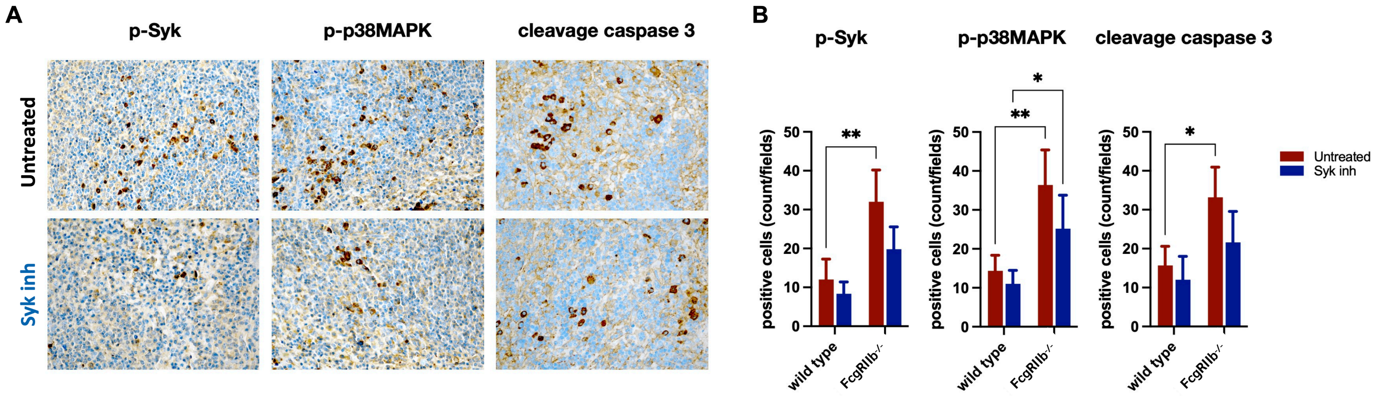

Supplement Figure 7: Immunohistochemistry of spleen from WT mice administration with and without Syk inhibitor (R788) indicated Syk activation, p38MAPK activation and cleavage caspase-3 (A) as compared to FcγRIIb$^{-/-}$ (n=5 mice for all groups). *; significant difference ($p<0.05$).
