# Supplementary material for: Syk inhibitor attenuates lupus in FcγRIIb−/− mice through the Inhibition of DNA extracellular traps from macrophages and neutrophils via p38MAPK-dependent pathway
Source: Cell Death Discov. 2025 Feb 17;11:63. doi: 10.1038/s41420-025-02342-x (PMC11832894; doi:10.1038/s41420-025-02342-x)
Supplement: Supplementary file 3 — Supplementation Materials and Methods [file 41420_2025_2342_MOESM3_ESM.pdf]

Materials and Methods

Mice and in vivo animal experiment

The animal protocols were approved by the Faculty of Medicine, Chulalongkorn University (No.2391019), and followed the National Institutes of Health (NIH) criteria. FcγRIIb$^{-/-}$ mice (C57BL/6 background) were provided by Dr Silvia Bolland (National Institute of Allergy and Infectious Diseases; NIAID, NIH). Wild type (WT) C57BL/6 mice were purchased from Nomura Siam International. The sample size was calculated with power analysis with 1.5 of effect size f and 0.95 of power goal, which has been show at least 4 mice/group. The mice were selected from similar age of parent, same age, and using only female. Both 40-wk-old mice (WT and FcγRIIb$^{-/-}$) were randomly divided into two groups (5 mice/group), which are the control group and Syk treatment group, respectively. Syk inhibitor (R788, fostamatinib disodium, Cat. S2206, Selleckchem, US) at 25 mg/kg/dose was orally administrated in 2 separate groups every 7 days for 4 weeks. Blood collection through the tail vein was performed 1 day before Syk inhibitor treatment as a baseline and compared progression of Syk inhibitor oral administration in every week. At the last week of the experiment, all mice were sacrificed, and blood was collected by cardiac puncture to explore lupus characteristics and leaky gut for comparing pre- and post-Syk inhibitor treatment. All experimented mice were blinding to the experimental examiner.

Lupus characteristics and leaky-gut measurement

For lupus characteristics, serum anti-dsDNA, proteinuria, glomerular pathology, and glomerular immune 10 complex deposition were detected. Briefly, serum anti-dsDNA was measured by ELISA-based measurements (Invitrogen). Bicinchoninic acid assay (PIERCE BCA protein assay kit, Thermo Fisher Scientific) was used to measure proteinuria. Serum endotoxin, BG, and FITC-dextran were applied for leaky gut measurement that evaluated endotoxin by the HEK-Blue™ TLR4 reporter cells (InvivoGen, rep-lps2) and 1,3-β-D-glucan by the Fungitell assay (Associates of Cape Cod) according to the manufacturer's instructions. The gut permeability marker FITC-dextran (molecular weight 4.4 kDa) (Sigma-Aldrich) at 50 mg/mL was orally administered to mice. Serum FITC-dextran was determined by fluorospectrometric, 3 h later administered and without FITC-dextran administration was used as background.

Bone marrow-derived macrophage (BMDM) and neutrophil isolation

The macrophages were derived from bone marrow (BM). Briefly, femur BM cells were obtained by centrifugation at 1,500 rpm for 10 min at 4°C. The BM cells were then incubated for 7 days in completed culture medium (DMEM media (Hyclone) with 10% (v/v) fetal bovine serum (FBS) (Hyclone), 5% horse serum (Hyclone), 1% (w/v) sodium pyruvate (Hyclone), 1% (w/v) HEPES (Hyclone), 100U/ml penicillin (Gibco) with 20% (v/v) L929 condition media (LCM) supplemented as a source of macrophage-derived growth factors (L929, ATCC, CCL-1). BMDMs were harvested with cold phosphate buffer solution (PBS), and the confirming phenotype with anti-F4/80 and anti-Ly6G antibody staining (Cat. 123110, BioLegend; cat. 551460, BD Biosciences) by flow cytometry. Analysis and graphical output were performed using FlowJo™ software (BD Tree Star).

Neutrophils from Bone marrow were isolated by EasySep™ mouse neutrophil enrichment kit performed through EasySep™ magnet (Stemcell technologies), by negative selection according to the manufacturer's protocol. To verify the phenotype of neutrophil by staining with anti-F4/80 and anti-Ly6G antibody (Cat. 123110, BioLegend; cat. 551460, BD Biosciences) by flow cytometry. Analysis and graphical output were performed using FlowJo™ software (BD Tree Star).

Cell culture and stimulation

BMDMs (2.5 x10^5 cells) and isolated neutrophils (2.5 x10^5 cells) were maintained in DMEM or RPMI 1640 medium containing 10% FBS at 37°C with 5% CO_2 incubator. For inhibitory studies, both cells were pre-incubated with Syk inhibitor (R406, Cat. S1533, Selleckchem, US) at concentrations of 100 nM and 200 nM or p38MAPK inhibitor (Adezmapimod, Cat. SB203580, Selleckchem, US) at concentrations 2 uM and 4 uM for 1 hour before stimulating with stimulators. After pre-incubation with inhibitor, cells were stimulated with a combination of LPS 100 ng/ml (Lipopolysaccharides from Escherichia coli O26:B6, MERCK) and whole glucan particle 100 ug/ml (WGP, Saccharomyces cerevisiae, Invivogen, SD) for optimal times to generate cellular activation in each cell.

FcγRIIb receptor quantification and macrophage polarization

All experiments to quantify the expression were performed by flow cytometry which were analyzed with FlowJo solfware (FlowJo, LLC). For measuring the effect of either stimulator or inhibitor to surface protein expression in vitro, the BMDMs after stimulation with or without inhibitor were measured the levels of FcγRIIb receptor expression (PE conjugated anti CD32b antibody, Cat. 12-0321-82, eBioscience), while the polarization of BMDMs were performed by measurement of CD86 and CD206 expression (FITC conjugated anti-CD86 antibody, Cat. 105006; APC conjugated anti-CD206 antibody, Cat. 141708, BioLegend), respectively. For measuring the effect of inhibitor to macrophage polarization in vivo, spleenic macrophages were identified by expressions of F4/80 and CD11b (Cat. 123110, BioLegend; cat. 552850, BD Biosciences) and the M1 polarization was performed by elevation of CD86 (Cat. 105006, BioLegend).

Cytotoxicity screening and cell viability assay

Both cells proceeded as cell culture conditions (untreated and treated with LPS and WGP conditions after pre-incubation in various concentrations of Syk inhibitor and p38MAPK inhibitor) for further 24 hours. MTS assay (Promega) was performed following the manufacturer's protocol. In the apoptosis assay, treated conditions were explained above of $1x10^5$ cells and controls (negative control; untreated condition, positive control; cycloheximide treated cells), which were collected and washed by PBS 1 time before being stained. Early apoptosis to late apoptosis/necrosis in the detected cell populations was assessed using Annexin V-APC and Propidium Iodide Apoptosis Detection Kit I (BD Bioscience). Stained cells were then analyzed on a flow cytometer (FACsAria II, BD), analyzing 20,000 events per sample. The gating strategy was set up according to FMO control for all antibodies. Analysis and graphical output were performed using FlowJo™ software (Tree Star, BD).

Macrophage and neutrophil-derived extracellular traps

MET and NET formation are determined after primed cell ($2.5x10^5$ cells) with each inhibitor on a coverslip, then stimulated with a combination of LPS (100 ng/ml) and WGP (100 ug/ml) for 24 hours (for BMDMs) and 6 hours (for neutrophils), respectively. The cells on coverslips were fixed with 4% paraformaldehyde in 1xPBS and blocked for 30 min. with 1xTBS with 2% bovine serum albumin. ET formation was detected using immunofluorescence staining against histone H3 citrullination (CitH3) using rabbit anti-histone H3 in dilution 1:300 (citrulline R2+R8+R17, ab5103; citrulline R26, ab212082, Abcam), subsequently with secondary antibody (goat anti-rabbit IgG H&L conjugated Alexa fluor488, ab150077, Abcam). DAPI (1ug/mL) was used to detect DNA. ET identification was observed by fluorescence microscope (Olympus, Japan). The analysis used ten representative pictures from each condition containing more than 20 cells.

ELISA

The supernatants collected from each experiment were analyzed with a quantitative sandwich enzyme immunoassay technique according to the manufacturer's instructions (Invitrogen, US) for measuring TNFa, IL-6, and IL-10 levels. Moreover, measuring citH3 levels in the supernatant was performed by ELISA using capture antibody against anti-histone H3 citrulline and detection antibody; HRP conjugated anti-histone H3 antibody (Cayman, US).

RNA sequencing analysis

The influence of a Syk inhibitor on $Fc\gamma RIIb^{-/-}$ and WT BMDMs in non-stimulated and stimulated conditions as indicated were extracted RNA using an RNAeasy Mini Kit (Qiagen, Germany) following the manufacturer's protocol. The RNA sequencing was performed and analyzed by BGI sequencing center (Hong Kong). Subsequent steps were carried out using Agilent 2100 Bioanalyzer (Agilent RNA 6000 Nano Kit) to check RNA quality, RNA integrity number (RIN scores >7.0 and 28S/18S >1), and fragment length distribution. The RNA-Seq data were mapped onto the reference genome (mm10_UCSC_20180117), followed by novel gene prediction, SNP & INDEL calling, and gene-splicing detection. After that, DEGs (differentially expressed genes) were used to identify between samples and do clustering analysis and functional annotations. For gene expression analysis, clean reads were mapped to reference using Bowtie2 (53), and then calculate gene expression level with RSEM (54) a software package for estimating gene and isoform expression levels from RNA-Seq data. Calculate Pearson correlation between all samples using cor, perform hierarchical clustering between all samples using hclust, and draw the diagrams with functions of R. For detection of DEGs, DEGs were detected with DEseq2 as requested. DEseq2 is based on the negative binomial distribution, performed as described (55). Identify the DEGs between samples or groups based on the gene expression level. With the KEGG annotation result, DEGs

were classified according to official classification and performed pathway functional enrichment using phyper, a function of R. false discovery rate (FDR) was calculated for each p-value, in general, the terms which FDR not larger than 0.01 are defined as significantly enriched. The datasets generated or analyzed for this study were deposited in a public database and can be found under the SRA: PRJNA1133910.

Western blot analysis

After pre-treated cells ($2.5x10^6$ cells) as described above were lysed with RIPA lysis buffer (Thermo Scientific^{TM}) supplemented with protease and phosphatase inhibitors (ab201111, Abcam). Protein concentrations were measured by bicinchoninic acid assay (PIERCE BCA protein assay kit, Thermo Fisher Scientific). The 20 ug/ml of protein/sample were separated on SDS-polyacrylamide gel electrophoresis and electroblotted onto a nitrocellulose membrane 0.22 um (Bio-Rad). Antibodies for western blotting were used as recommended concentration following to manufacturer's instruction (Cell Signaling Technology); rabbit anti-Syk (1:1000, Cat. 13198), rabbit anti-phospho-Syk (1:1000, Cat. 2717), rabbit anti-p38 MAPK (1:1000, Cat. 9212), rabbit anti-phosphor-p38 MAPK (1:1000, Cat. 9211), rabbit anti-p44/42 MAPK (Erk1/2) (1:1000, Cat. 9102), rabbit anti-phosphor-p44/42 MAPK (1:1000, Cat. 9101), rabbit anti-SAPK/JNK (1:1000, Cat. 9252), rabbit anti-phospho-SAPK/JNK (1:1000, Cat. 4668). The secondary antibodies including rabbit anti-histone H3 (ab5103; 1:1000, ab212082; 1:500, Abcam) and rabbit polyclonal anti-GAPDH (1:1000, Cat. 5174), anti-rabbit IgG (1:5000, Cat. 7074). Membrane images were captured using the ImageQuantTM LAS 500 (GE Healthcare, Life Sciences) and analyzed with the imageLab software (Bio-Rad, US).

Histology and immunohistochemistry

The collected kidney, spleen, and intestine were fixed with 4% paraformaldehyde for 72h following paraffin embedding, slide section, and staining with hematoxylin and eosin (H&E) to observe and measure the injury score. The results were performed at ×200 magnification in 5 random fields for each model. For immunohistochemistry, the sections were blocked by 2%BSA to reduce non-specific binding. The primary antibody (Cell Signaling Technology) rabbit anti-phospho-Syk (1:300, Cat. 2717) or rabbit anti-phospho-p38 MAPK (1:300, Cat. 9211), for detecting protein expression, and anti-cleavage caspase-3 (1:400, Cat. 9661) for measuring apoptosis) were incubated overnight at 4 °C. Subsequently, secondary antibodies (matching species to primary antibody), streptavidin peroxidase (SP) complex were incubated at 37 °C for 30 min. and stained with DAB reagent. The results were captured by a bright field microscope (Nikon Eclipse I with Nikon software, Japan) and performed at ×200 magnification in 5 random fields for each model.

Parallelly, freezing spleen and kidney were embedded in optimal cutting temperature (O.C.T., Sakura Finetek, Japan) following cryosection and acetone fixed. After, the sections were blocked non-specific binding by 2%BSA (RT, 30 min.), primary antibodies (rabbit anti-phospho-Syk (1:300, Cat. 2717, Cell Signaling Technology), rabbit anti-histone H3 in dilution 1:300 (citrulline R2+R8+R17, ab5103; citrulline R26, ab212082, Abcam) and anti-F4/80 (1:300, ab6640, Abcam) were incubated overnight at 4 °C. The Secondary antibodies (donkey anti-rabbit Alexa fluor647, ab150075; goat anti-mouse Alexa fluor488, ab150113, Abcam) were incubated 90 min before nuclei were stained by DAPI. Immunocomplex deposition in the tissue was detected by Goat anti-mouse IgG conjugated Alexa fluor 488 (1:200, ab150113, Abcam) for 2 h. Observed the slides under a confocal fluorescence microscope (NIKON ECLIPSE C1), and images were collected.

Quantification and Statistical analysis

All experiments were performed in triplicate and the experimenters, who treat and collect the samples from mice, have randomly assigned the samples to other experimenters for blind evaluation. The results were analyzed and qualified by two experimenters. All data were analyzed by Statistical Package for Social Sciences software (SPSS 22.0, IBM Corporation, Armonk, NY, USA) and visualized using Graph Pad Prism version 7.0 software (La Jolla, CA, USA). Results are presented as mean ± standard deviation (SD) or median with internal quartile range (IQR). Parametric or non-parametric statistic tests were applied depend on data distribution. Statistical differences among groups were examined using the unpaired Student's t-test or Man-Whitney U test for analyzing independent 2 groups and the one-way ANOVA or Kruskal Wallis test with multiple comparison test to analyze experiments with more than 2 groups, respectively, strictly followed assumptions of the tests (e.g. normal distribution, variance estimation, and data transformation). Differences with a p-value < 0.05 were statistically significant.
